# Editorial: The Interplay Between Immune Activation and Cardiovascular Disease During Infection, Autoimmunity and Aging: The Role of T Cells

**DOI:** 10.3389/fimmu.2021.719517

**Published:** 2021-06-24

**Authors:** Eva Reali, Sara Ferrando-Martinez, Marta Catalfamo

**Affiliations:** ^1^ Department of Biotechnology and Biosciences, University of Milano-Bicocca, Milan, Italy; ^2^ NeoImmuneTech, Inc., Rockville, MD, United States; ^3^ Department of Microbiology and Immunology, Georgetown University School of Medicine, Washington, DC, United States

**Keywords:** T cells, cardiovascular disease, infection, aging, autoimmunity

## Introduction

Chronic activation of cells of the immune system including T cells and systemic inflammation are well known risk factors for cardiovascular disease (CVD). Many human pathological conditions including viral infections, autoimmune diseases and aging are recognized drivers of increased risk of CVD. Among viral infections, Cytomegalovirus (CMV) infection is a contributing risk element to the existing traditional risk factors of atherogenesis; Influenza infection is correlated with increased the risk of cardiovascular events leading to deaths and HIV infection is an independent predictor of cardiovascular risk. The pandemic of SARS-COV2 infection showed that the severe presentation of the disease manifests with vascular damage and cardiovascular events. Autoimmune and chronic inflammatory diseases, including systemic lupus erythematosus (SLE), rheumatoid arthritis (RA) and psoriatic disease are also associated with cardiovascular disease. Lastly, in adults over 65 years, the accumulation of age-related phenotypic and functional alterations in immune cells parallels with a decline of the cardiovascular system with an increased incidence of cardiovascular disease.

The mechanisms behind are not well defined, and while the role of innate immune cells has been established, the involvement of T cells in promoting vascular pathology and cardiovascular disease has emerged more recently (1). Chronic systemic inflammation and increased circulating levels of cytokines and chemokines can indeed contribute to vascular damage by promoting endothelial cell activation and oxidative stress thus linking to the increased risk of CVD (2, 3). Activation of endothelial cells promotes recruitment of circulating immune cells including T cells that will be activated and differentiate into distinct effector cells contributing to the pathology of the disease (4–6).

Endothelial cells in this context have also been proposed to act as “semiprofessional” antigen presenting cells (APC) presenting antigens and providing several costimulatory signals to T cells leading to T cell activation especially at sites defined as endothelium-dependent microvascular reactivity sites by Laser Doppler Flowmetry Assessment (7). A milestone in the understanding the role of T cells in promoting vascular inflammation has been reached with the characterization of the immune cell infiltrate in human atherosclerotic plaque by scRNA-seq technology which defined the main subsets of T cells in atherosclerosis (8). This data paved the way for further investigations about the role of T cells as a putative mechanistic link in pathologies associated with an increased risk of CVD. The proposed mechanisms by which T cells contribute to the pathology of the disease include dysregulated T helper and CD8 T cell function, expansion of terminally differentiated cytotoxic effectors CD4^+^CD28^-^ T cells and impaired Tregs function.

This Research Topic has the aim to provide an overview of the latest advances in the study of the role of T cell activation and endothelial inflammation in cardiovascular risk and disease in the context of infection, autoimmunity and aging. The Research Topic highlights the emerging common and distinctive features of the putative immune mechanistic links between the pathophysiological conditions and the associated cardiovascular disease. The Research Topic comprises 11 articles, original research articles, 5 review and one systematic review and was divided into 3 sections.

1. Endothelial inflammation and T cell activation in CVD associated with infection.
2. T cell mechanisms involved in CVD associated with autoimmune diseases.
3. The immunology of cardiovascular disease during aging.

## T Cell Activation, Endothelial Inflammation in the Setting of Infection and Its Impact In CVD Risk

Platelets play an important role in the immunity against pathogens, and in the setting of chronic inflammation they can promote vascular damage, atherogenesis, and thrombosis. In this review by Zamora et al. the authors focus the attention in the role of platelets (PLTs), systemic inflammation and CVD. This article highlights the dual role of platelets in promoting both pro-and anti-inflammatory processes and underlines their capability to interact with leukocytes and endothelial cells. They discuss that this mechanism contributes to systemic inflammation by favoring the arrest of leukocytes on endothelium, the production of inflammatory cytokines and NETs formation. On the other hand, the binding of PLTs to leukocytes decreases the inflammatory response, participating in the resolution of thrombo-inflammation. The authors also mention the use of platelet to lymphocyte ratio as an emerging biomarker of systemic inflammation and cardiovascular outcomes.

In this complex interaction between cells of the immune system and the vasculature, Ferrari et al. illustrate the importance of purinergic signaling in modulating pro- and anti-atherogenic responses, particularly in T cells and macrophages. Extracellular nucleotides (ATP, ADP, UTP and UDP) and nucleosides such as adenosine are known to be involved in the onset of pathologic states including blood hypercoagulability, thrombosis and atherosclerosis. Nucleotides and nucleosides signal through P2 and P1 receptors respectively modulating a wide array of cell and tissue functions. Particularly, extracellular ATP is an important activator of NLRP3 inflammasome, and the lack of the purinergic receptor P2X7 (P2 receptor) signaling in mouse model of atherosclerosis showed decreased disease development. Therefore, these data strengh the importance of investigating purinergic signaling in inflammasome activation and atherosclerosis.

In the setting of chronic infections immune activation and systemic inflammation is also associated with increased cardiovascular risk. People with HIV (PWH) infection have twice the risk of cardiovascular disease compared to the general population (9). Endothelial inflammation and injury play a critical role in the pathogenesis of cardiovascular risk/disease. The homeostasis, maintenance and repair of endothelial cells is mediated in part by bone marrow-derived endothelial cell progenitors and other cell progenitors from the hematopoietic origin participate in this process. In addition, a subset of T cells, angiogenic T cells (T_angs_) can support the proliferation and differentiation of endothelial progenitor cells. In this manuscript, Zhu et al. investigated the role of HIV driven immune activation in the process of endothelial repair. They found two subsets of circulating progenitors LIN4^-^CD45^-^CD34^+^ and LIN4^-^CD45^dim^CD34^+^ in PWH, and showed that the phenotype but not frequencies was associated with biomarkers of inflammation. Importantly, CD8 T_ang_ cells express the chemokine receptor CX3CR1 suggesting the recruitment of pro-inflammatory T cells to the sites of endothelial injury. This data provides a new tool to better address the impact of HIV infection in endothelial inflammation and repair and the players involved in the process.

In the review by Gopal R et al., the authors discuss in depth the increased risk of cardiovascular morbidity and mortality during influenza infection. They examine the immune mechanisms driving the pathogenesis and cardiovascular risk, and they provide striking similarities and differences between the epidemiological and pathogenic mechanisms involved in cardiovascular events associated with coronavirus disease 2019 (COVID-19) and influenza infection. The authors highlight the pathology induced by either direct viral infection or indirect tissue damage due to the inflammatory cytokine storm in these infections, and the impact of these factors when combined with underlying conditions such as atherosclerosis. Interestingly, while influenza virus triggers type I and type II IFN responses, the induction of IFNs in the setting of SARS-CoV2 infection, in respiratory epithelial cells is low and this may contribute to the increase viral replication and immunopathology in the lung. Lastly, the authors discuss the potential role of the dysregulation of the immune responses and the increased risk of cardiovascular events.

The original article by Huaman et al. investigates the role of *Mycobacterium tuberculosis* infection and risk of cardiovascular disease. Studies had indicated that people with a history of tuberculosis disease, and latent tuberculosis infection have higher risk of coronary artery stenosis and myocardial infarction. To address the mechanisms behind these associations, they use the *Ldlr* ^-/-^ mice, a murine model of atherosclerosis. They evaluate the impact of *M. bovis* BCG infection in exacerbating disease. They found that despite no significant differences in plasma cholesterol or triglyceride levels between the infected and uninfected mice fed with western diet, infection induced immunological changes in T cells (increase CD4/CD8 ratio) and monocytes (Ly6C low non-classical monocytes) that were associated with increased atherosclerotic lesion formation in the aorta. These data propose a mechanistic model in which mycobacterial infection is capable of enhancing atherosclerosis development.

## T Cell Mechanisms Involved in CVD Associated With Autoimmune Diseases

The role T cells in CVD associated with autoimmune and chronic inflammatory diseases is comprehensively summarized by the review article of this Research Topic by Schwartz et al. focused on evidence indicating that T cells are drivers of vascular inflammation in autoimmunity associated CVD. In particular it is considered the role of T cells in primary vasculitis and on three systemic autoimmune diseases: such as rheumatoid arthritis (RA), systemic lupus erythematosus (SLE), and psoriasis.

The authors first underline the role of T cells dysregulation in systemic autoimmunity supported by the association between early onset autoimmunity and mutations in FOXP3 and CTLA4 genes and single nucleotide polymorphisms (SNPs) in genes involved in T cell functions. They point out that autoimmune diseases are often paralleled by the presence of autoreactive T cells opening the concept that self-reactive T cells may represent the link between autoimmune disease and the risk of CVD in patients with RA, SLE and psoriasis. Accordingly, in rheumatoid arthritis, CD4 and CD8 T_EM_ cells are expanded and correlate with coronary artery calcification. In addition, expansion of CD4^+^CD28^-^ T cells are observed in these patients with preclinical atherosclerosis. Th17 subset on the other hand, is likely to play a major role in linking CVD and atherosclerosis in psoriasis. In addition, CD4 expressing CXCR3 have been shown to play a role in the migration to the arterial wall and induction of vascular inflammation in the setting of SLE. Interestingly Abatacept treatment in SLE patients decreases the expansion CD4^+^CD28^-^ T cells.

The role of T cells in vasculitis is further discussed in the review presented by Watanabe et al. focusing on large vessels vasculitis such as Giant Cell Arteritis (GCA) and Takayasu arteritis (TAK) that are characterized by an aggressive inflammation of arteries leading to hemorrhage and occlusions of the vessels. T cells play a major role in the pathogenesis of these two diseases which have long been considered largely overlapping. The authors underline the substantial differences between giant cells arthritis and Takayasu arteritis in both genetic association and the contribution of CD4 and CD8 T cell subsets in the pathology of the disease. Moreover, the authors discuss the considerable resistance to treatment underlining the importance to evaluate signaling pathways involved and their redundancy to therapeutically target the most relevant mechanism in each disease/patients. These include the sustained activation of mTORC1 and the JAK STAT pathway, that leads to a sustained immune responses in arterial wall, an immune privileged site that is intolerant to damage.

The involvement of T cells in CVD is further discussed in the manuscript by Bortoluzzi et al. Specifically, the study is focused on angiogenic T cells and CD4^+^ and CD8^+^CD28^-^ T cell subsets in patients with Systemic Lupus Erythematosus and CVD. Angiogenic T (T_ang_) cells are a subset of T cells (CD3^+^CD31^+^CXCR4^+^) that promotes vasculogenesis and their characterization represents a promising field of research in cardiovascular medicine. Through the secretion of proangiogenic factors T_ang_ cells exert a critical role in the formation of colonies of endothelial progenitor cells (EPCs) as well as in their differentiation and function with a resulting protective role in the context of CVD. Within T_ang_ subset, in SLE patients, there was a significant expansion of a subpopulation with immunosenescent characteristics such as the loss of the costimulatory molecule CD28, required for T cell activation, survival and proliferation. This exhausted subset of T_ang_ correlated with SLE disease activity index- and was inversely related to T_ang_ cells percentage. The data of this study definitely point out the importance of reinforcing the knowledge of loss of CD28 in the T_ang_ compartment in relation to cardiovascular risk and lower percentage of endothelial precursor cells in SLE patients.

Further Chernomordik et al. analyze the role of autoreactive T cells in CVD. They analyzed the presence of reactive T cells to the cathelicidin antimicrobial peptide LL-37 in patients with acute coronary syndrome. In a murine model of atherosclerosis (ApoE^-/-^) immunized with the mouse orthologue of LL-37, mCRAMP, they present evidence of role of LL-37 reactive T cells in the pathology of the disease. LL-37 is a major driver in the initial phase of psoriasis pathogenesis and a target of self-reactive T cells identified in patients. LL-37 is also present in human atherosclerotic plaques, and it associates with platelet activation and induction of thrombosis. Therefore, the authors investigated these molecules as a potential linker between the innate immunity activation in chronic inflammation with autoreactive T cell generation and cardiovascular disease. The study shows that LL-37 stimulation of PBMCs from patients with acute coronary syndrome induced the persistence of CD8 T_EM_ cell response compared to PBMCs from patients with stable coronary artery disease. However, in ApoE deficient mouse, adoptive transfer of T cells from mice immunized with mouse orthologue of LL-37 mCRAMP was associated with smaller atherosclerotic aortic plaque area and absence of aortic sinus plaque calcification. This work represents a starting point of an interesting future perspective that could reveal key aspects of the mechanistic link between DAMP-mediated priming of a chronic inflammatory/autoreactive response and cardiovascular events.

The study by Zhao R. et al. analyzes the expression of Aryl hydrocarbon receptor (AHR) in peripheral blood mononuclear cells (PBMCs) from patients with Type-2 diabetes (T2D) and metabolically healthy obesity (MHO). AHR is a ligand-activated transcription factor regulated by small molecules derived from diet, metabolism or xenobiotics and critical transcription factor determining the lineage commitment of pro-inflammatory Th17 and Th22. Its ligands have been epidemiologically linked to obesity and type-2 diabetes. The results reported in this research article indicate that the expression of AHR mRNA in PBMCs was increased in patients compared to healthy subjects and correlated with plasma levels of IL-17 and IL-22 in metabolically healthy obese and T2D patients. Correlation was also observed with serum hsCRP levels and with the index of insulin resistance. The authors highlight a potential role of AHR in the interplay between metabolism, inflammatory status and the development of obesity and T2D, and hypothesize a role for AHR as a sensor involved in metabolic stress response.

## The Immunology of Cardiovascular Disease During Aging

Aging involves highly variable age-related changes in all organs, tissues and cells, and confers vulnerability to different stressors and diseases. Of particular interest are the age-related changes in the immune and cardiovascular systems. While aging of the cardiovascular system is associated with highly prevalent age-related diseases, a growing body of knowledge has shown that the aging of the immune system plays an active role of cardiovascular health.

In this Research Topic, Delgobo et al. analyze the relationship between the age-related accumulation of terminally differentiated CD4 T cells and myocardial inflammation. Using a xenograft mouse model this study elegantly shows that human naïve CD4 T cells, when transferred to immune-depleted mice, undergo homeostatic proliferation and differentiation of effector cells. Together with the accumulation of differentiated and terminally differentiated CD4 T cells, the authors observed an increase of effector CD4 T cell infiltration, monocytes, macrophages and dendritic cells infiltrating the myocardium, a phenotype that recapitulates age-related changes in elderly individuals. This interesting study suggests that the age-related accumulation of terminally differentiated CD4 T cells could play a critical role in promoting myocardial alterations in the aging human heart.

However, while trying to understand the interconnection and influence of the age-related changes of the immune system with the incidence and prevalence of cardiovascular diseases in the elderly, we should be mindful of the high heterogeneity that exists among all studies focusing on immune system aging. To better understand this limitation, Rodriguez et al. performed a meta-analysis to determine the best set of markers to define immunosenescent cells. Strikingly, they observed that the lack of consensus not only on markers, but also on techniques and the high risk of bias did not allow for the identification of this “validated” phenotype. This result strongly suggests that active efforts towards identifying standard operating procedures (SOPs) are needed if we ever want to understand such a complex process as it is the aging of the immune system and its crucial role in the aging process of all other systems, including cardiovascular aging.

## Concluding Remarks

This Research Topic highlights T cell mediated mechanisms involved in the interplay between a dysregulated immune T cell activation such as those observed in the setting of an acute, chronic infection, autoimmune diseases, aging and cardiovascular risk/disease. While the pathogenesis of these human diseases is unique, the integrated view provided in this collection of manuscripts delineate common features linking T cell activation and increased CVD risk. Several CD4 and CD8 T cell subsets seems to be involved participating in the disease pathology promoting endothelial inflammation and injury, progression of atherosclerotic plaques and infiltration of myocardium. Distinct subsets of T cells including CD4 T_EM_ cells with Th1 function and those with a CD4^+^CD28_null_ T cell phenotype have been described. T cells producing IFNγ and pro-inflammatory cytokines, and cytotoxic function are shown to be expanded in patients with CVD in lupus and other diseases and correlated with disease severity. The CD8 T cell subset is also involved and its role appears to be relevant in advanced phases of CVD such as atherosclerosis, in which CD8 T cells are present mainly in fibrous cap areas at higher number than CD4 T cells. These T cells have cytotoxic activity and secrete pro-inflammatory cytokines driving the progression and instability of the lesions. Finally, the emerging role of T cells in vasculogenesis highlights another important function of T cells. This angiogenic T cell subset or T_ang_ subpopulation is enlightened as it is involved in vascular repair processes promoting new vessel formation, and new insights are provided into their role in pathogenesis or vascular disease. In addition, T helper cells expressing AHR, a putative sensor for environmental and metabolic stressors emerge as a potential subset that could link metabolic dysregulation with systemic chronic systemic inflammation in patients with Type-2 diabetes (T2D) and metabolically healthy obesity (MHO).

Overall, this Research Topic highlights the role of T cell immune activation in cardiovascular disease suggesting an important role of T cells in the homeostasis of the vascular and cardiac system, and when dysregulated can contribute to the pathology of the disease. These emerging concepts offer new interesting avenues of investigation of the role of T cell activation in health and disease, and identify novel diagnostic and prognostic biomarkers of cardiovascular risk in pathologies characterized by a dysregulated immune T cell activation.

## Author Contributions

ER, SFM and MC wrote the article. All authors contributed to the article and approved the submitted version.

## Funding

MC is supported by: The National Institute of Allergy and Infectious Diseases of the National Institutes of Health under award number NIH R01AI145549-02. Leidos Biomedical Research, Inc. has been funded in whole or in part with federal funds from the National Cancer Institute, NIH, under Contract HHSN261200800001E. The content of this publication does not necessarily reflect the views or policies of the Department of Health and Human Services, nor does mention of trade names, commercial products, or organizations imply endorsement by the U.S. Government. MC is supported in part by the District of Columbia Center for AIDS Research, an NIH funded program (P30AI117970). The content is solely the responsibility of the authors and does not necessarily represent the official views of the NIH.

## Conflict of Interest

SFM was employed by NeoImmuneTech, Inc.

The remaining authors declare that the research was conducted in the absence of any commercial or financial relationships that could be construed as a potential conflict of interest.
